# Recent advances in understanding and managing infectious diseases in solid organ transplant recipients

**DOI:** 10.12688/f1000research.14262.1

**Published:** 2018-05-24

**Authors:** Claire Aguilar, Shahid Husain, Olivier Lortholary

**Affiliations:** 1 Division of Infectious Diseases, Multi-Organ Transplant Program, Department of Medicine, University of Toronto, University Health Network, Toronto, ON, Canada; 2 Necker Pasteur Center for Infectious Diseases and Tropical Medicine, Paris Descartes University, IHU Imagine, Paris, France

**Keywords:** donor-derived infections, Hyperammonemia syndrome, immune monitoring, vaccine

## Abstract

**Background:** Undergoing solid organ transplantation (SOT) exposes the recipient to various infectious risks, including possible transmission of pathogen by the transplanted organ, post-surgical infections, reactivation of latent pathogens, or novel infections.

**Recent advances:** In the last few years, the emergence of Zika virus has raised concerns in the transplant community. Few cases have been described in SOT patients, and these were associated mainly with moderate disease and favorable outcome; the notable exception is a recent case of fatal meningo-encephalopathy in a heart transplant recipient. Because of the advances in treating hepatitis C, several teams recently started to use organs from hepatitis C-positive donors. The worldwide increasing incidence of multidrug-resistant pathogens, as well as the increasing incidence of *Clostridioides* *difficile* infection, is of particular concern in SOT patients. In the field of mycology, the main recent therapeutic advance is the availability of isavuconazole for the treatment of invasive aspergillosis and mucormycosis. This drug has the advantage of minimal interaction with calcineurin inhibitors. Regarding the viral reactivations occurring after transplant, cytomegalovirus (CMV) infection is still a significant issue in SOT patients. The management of resistant CMV remains particularly difficult. The approval of letermovir, albeit in bone marrow transplantation, and the therapeutic trial of maribavir bring a ray of hope. Another advancement in management of post-transplant infections is the development of *in vitro* tests evaluating pathogen-specific immune response, such as immunodiagnostics for CMV and, more recently, tests for monitoring immunity against BK virus.

**Conclusion: **The increasing number of organ transplantations, the use of newer immunosuppressive drugs, and high-risk donors continue to define the landscape of transplant infectious diseases in the current era.

## Introduction

Solid organ transplantation (SOT) is a life-saving procedure. The advances in surgical techniques, as well as the application of better preventive and management strategies for organ rejection, have led to improved outcomes ^1– 3^. Conversely, the use of immunosuppressive medications may result in high infection risk and significant morbidity and mortality. Ongoing assessment of the epidemiology of those infections and evaluation of the modalities of prevention and treatment are critical to further improve outcomes in SOT recipients.

In this review, we will focus on recent advances in the understanding and management of infections in SOT patients, which can occur at different phases of the transplant process. We will initially describe the emergence of some newer donor-derived infections, followed by the summary of new drugs available for treating infections in SOT patients, prior to discussing the role of immune monitoring in the management of infections, and finally we will highlight the potential role for the newer vaccines.

## Donor-derived infections

### The fear of Zika virus…

Since 2015, the Zika virus emerged initially in South America and then in the Caribbean, Central America, and the southern US, raising concerns in the transplant community about the risk of transmission through organ donation and possible risks of severe disease in SOT recipients ^4, 5^. Zika virus belongs to the flavivirus family. In immunocompetent individuals, Zika virus can induce viral illness with symptoms similar to those of other arbovirus infections but is in fact asymptomatic in the majority of cases. It has also been associated with neurological manifestations such as Guillain-Barré syndrome and meningo-encephalitis. Besides transmission by mosquito bite, known routes of transmission are sexual, blood-derived, or materno-fetal. It has also been shown that in immunocompetent patients, Zika virus can persist in semen and saliva for several weeks after clearance of viremia. As such, potential persistence of Zika virus in organs needs further investigation. Zika can also be acquired after transplantation if the recipient lives in or travels to an endemic area. In 2016, Nogueira *et al*. published a case series of four SOT recipients (two kidney and two liver transplant recipients) in Brazil who presented with viral symptoms and tested positive for Zika virus ^6^. The time from transplant to positive testing was 43 to 590 days, and the testing of donors was not available. All patients experienced complications such as graft dysfunction or bacterial infections, although it is hard to know what role the Zika infection had in the symptoms. None of the patients had neurological symptoms, and all survived ^6^. However, in 2017, Schwartzmann *et al*. reported a case of fatal meningo-encephalitis due to Zika virus in a heart transplant recipient ^7^. Zika virus should be considered a possible cause of meningo-encephalitis in patients living in endemic areas as well as in patients who travelled to those areas. In immunocompetent patients, cases with few weeks between the onset of infection and neurological symptoms have been reported, warranting caution in SOT patient populations in which little is known about Zika virus infection ^8^. Recent transplant guidelines suggest performing nucleic acid testing for donors who have recently travelled in endemic areas and to exclude the organ if the donor is viremic ^9^. However, the low number of reported cases so far and the relative paucity of data on post-transplant infection question the validity of those recommendations. More studies are required to define the best strategies for donor screening, diagnosis, and management of Zika virus infections in SOT recipients.

### … the surprising *Ureaplasma spp*.

In the last few years, an unexpected infection, probably donor-derived, has been reported in lung transplant recipients. In this population, a rare but severe disease named hyperammonemia syndrome has been described since the ’90s ^10^. Hyperammonemia syndrome is characterized by elevated ammonia plasma levels in the early transplant period, complicated by neurologic symptoms, which can be fatal ^11^. In 2015, Bharat *et al*. reported several cases of hyperammonemia syndrome associated with the detection of *Ureaplasma urealyticum*, *Ureaplasma parvum*, or *Mycoplasma hominis* in respiratory samples and blood from lung transplant recipients ^12^. In 2017, Fernandez *et al*. reported another case with detection of *U. urealyticum* in both recipient and donor respiratory samples, suggesting a donor-derived infection ^13^ *.* The same team conducted a prospective study to assess the incidence of *Ureaplasma spp.* in a cohort of donors. They found that 4% of donors’ respiratory samples were positive for *Ureaplasma spp.* ^14^. Most of the positive samples were from young males, who had an aspiration event prior to death. Some centers routinely monitor ammonia levels in all lung transplant recipients during the first weeks post-transplantation and institute hemodialysis in cases of hyperammonemia along with antibiotic treatment directed against *Ureaplasma* and *Chlamydia*. Another strategy is to perform polymerase chain reaction for *Ureaplasma spp.* and *Mycoplasma spp.* in respiratory samples from donors and initiate therapy in the recipients if the donor sample is positive. However, these strategies need to be evaluated.

### … and a worrisome Candida infection

In 2017, the first case of donor-derived infection with *Candida auris* in a lung transplant recipient was reported in the US ^15^. *C. auris* is an emerging Candida species first described in 2009 in Japan and then in a dozen other countries ^16^. Whole-genome sequencing techniques identified four different clades with distinct geographical clustering, suggesting independent emergence in different areas. Isolates frequently exhibit high minimal inhibitory concentrations to antifungal drugs, and *C. auris* infections have been associated with poor outcomes ^17^. Moreover, the microbiological identification can be difficult. In the case reported by Azar *et al*. ^15^, the yeast was initially identified as *C. haemulonii*, which has been reported in several other cases of *C. auris* infection. The emergence of this multiply resistant Candida species warrants caution with regard to the identification of yeast in donor samples.

### A paradigm shift in donor-derived infections

A pressing issue in transplantation is the gap between the number of patients awaiting a transplantation and the number of organs available. In order to increase the pool of donors, retrieving organs from previously excluded donors is an emerging strategy. Several centers reported transplantation from HIV-positive donors to HIV-positive recipients ^18^ with favorable outcomes ^19, 20^. Moreover, the recent advances in antiviral therapies against hepatitis C have led to new perspectives in the field of transplantation, as those drugs have excellent efficacy and tolerance profiles, including in SOT recipients ^21^. In the last year, several centers have reported the use of organs from hepatitis C donors with detectable viremia at the time of transplant ^22^, and so far outcomes have been favorable with either monitoring of viral load triggering treatment ^23, 24^ or pre-emptive treatment ^25^.

## Infections acquired after transplantation: new treatments available

### Multidrug-resistant bacteria in solid organ transplantation

The global increase in antimicrobial resistance, which is a worldwide concern ^26^, is also particularly worrisome in the context of organ transplantation ^27^. SOT patients are highly exposed to the healthcare system, undergo different types of invasive procedures, and often require several courses of antibiotics. Few new antibiotics have been marketed in the last few years. Ceftolozane is a new beta-lactamin with anti- *Pseudomonas* activity, and the combination of ceftolozane-tazobactam has a broad spectrum, including *Enterobacteriacae* producing extended-spectrum beta-lactamase (ESBL). Ceftolozane-tazobactam, indicated mainly for multidrug-resistant *Pseudomonas* infections, has been approved by the US Food and Drug Administration (FDA) for the treatment of complicated intra-abdominal and urinary tract infections ^28, 29^. A recent retrospective study of 21 patients who received treatment for severe infections (pneumonia in 86% of cases) due to multidrug-resistant *Pseudomonas spp.* included eight SOT patients. Ceftolozane-tazobactam was well tolerated and was effective in 71% of patients. However, resistance to ceftolozane-tazobactam developed during treatment in three patients ^30^. Another interesting antibacterial agent is the association of ceftazidime with a new beta-lactamase inhibitor, avibactam, which inhibits the activity of some carbapenemases ^31^. In this context of increasing resistance, antimicrobial stewardship programs have developed tremendously in the last decade. Although the general practice is to provide treatment to SOT patients empirically with broad-spectrum antibiotics and to use prolonged duration of therapies, antimicrobial stewardship probably has a role to play in SOT recipients as well. So *et al*. conducted a retrospective analysis of antimicrobial prescriptions in an SOT population ^32^. A total of 176 audits were performed in 139 patients, and 30% of antimicrobial prescriptions were stewardship discordant ^32^. Several centers have now implemented dedicated stewardship programs ^33^.

### *Clostridioides difficile* 

The other consequence of broad-spectrum antibiotic use is the increase of *Clostridioides difficile* infections ^34^. A meta-analysis of published data in SOT recipients from 1994 to 2014 estimated the overall prevalence at 7.4%, and the recurrence rate was 19.7% ^35^. Interestingly, a recent case control study in Switzerland found an increased risk of graft loss in SOT patients with *C. difficile* infection ^36^. With regard to treatment, besides the use of antibiotics active against *C. difficile* (vancomycin, metronidazole, and fidaxomicin), other approaches are in development. Fecal transplantation has been used more and more in the last decade, and its efficacy has been established in immunocompetent patients to reduce the rate of recurrence of *C. difficile* colitis ^37^. However, the use in immunocompromised patients has been limited because of concerns about side effects and the sparse data in SOT patients ^38, 39^. Alraba *et al*. recently reported outcomes of 13 patients receiving fecal transplantation for recurrent *C. difficile* infection, including six SOT recipients. Although the fecal transplant was successful in six immunocompetent patients, three SOT patients failed ^40^. More recently, two monoclonal antibodies directed against *C. difficile* toxin A and toxin B—actoxumab and bezlotoxumab, respectively—have been evaluated in a double-blind, randomized placebo-controlled trial ^41^, including a cohort of 21.4% of immunocompromised hosts. In this study, use of bezlotoxumab was associated with a lower recurrence rate compared with placebo. The potential benefit of bezlotoxumab in SOT patients needs to be determined.

### Fungal infections

In the field of fungal infections, the major news in the last few years has been the approval of isavuconazole, a new triazole agent with broad activity, including *Aspergillus spp.* and mucorales. Its non-inferiority to voriconazole for the treatment of invasive aspergillosis has been established in a randomized clinical trial in patients with hematological malignancies ^42^. Its efficacy in cases of mucormycosis was suggested in a single-arm control trial involving a limited number of patients combined with a case control study ^43^ *.* Although isavuconazole was approved as first-line treatment of mucormycosis, liposomal amphotericin B remains a reference treatment in this indication. Interestingly, a prospective pilot study assessing the use of a high dose of liposomal amphotericin B, combined with surgery when feasible, showed a superior response rate at 12 weeks of treatment compared with the rates reported with isavuconazole. However, this study included only three SOT patients ^44^. The role of isavuconazole in prophylaxis and treatment of fungal infections in SOT patients needs to be determined. One advantage is the profile of tolerability of isavuconazole, which has less liver toxicity than voriconazole, and lack of nephrotoxicity, which can be an issue with liposomal amphotericin B. Like other triazoles, isavuconazole is an inhibitor of cytochrome P450 but inhibits only one isoenzyme compared with the three inhibited by voriconazole. Data about drug interactions with calcineurin inhibitors were initially reported in healthy volunteers ^45^ and showed a 1.3-fold increased exposure to cyclosporine, 2.3-fold increase to tacrolimus, and 1.8-fold increase to sirolimus. Rivosecchi *et al*. recently reported their experience in Pittsburgh, where a universal prophylaxis with isavuconazole has been established in all SOT patients after an outbreak of mucormycosis ^46^. The authors found that overall the changes of tacrolimus plasma concentrations induced by the co-administration of isavuconazole were mild, and a 1.3-fold decrease in tacrolimus dose was necessary to maintain the tacrolimus level. Interestingly, the changes in tacrolimus plasma concentrations were seen mostly in liver transplant recipients.

### Viral infections

Cytomegalovirus (CMV) infection remains a significant issue in SOT patients. Additionally, the management of infection with viruses resistant to first-line treatment (ganciclovir and valganciclovir) is particularly challenging, as alternative drugs (foscarnet and cidofovir) carry significant toxicities. As such, new drugs possessing a better toxicity profile are eagerly awaited in SOT patients. Maribavir is an inhibitor of UL97 viral kinase. In a study of liver transplant recipients, the use of maribavir 100 mg twice daily did not prevent CMV infections ^47, 48^. However, its efficacy for the treatment of refractory or resistant CMV disease in SOT patients has been reported with higher doses ^49, 50^. Occurrence of resistance has been reported in treatment with maribavir ^51^. A phase 3 trial for the treatment of refractory or resistant infection in transplant patients is ongoing. Letermovir, a novel non-nucleoside CMV inhibitor targeting the viral terminase complex, was approved by the FDA in 2017 for the prevention of CMV infection in bone marrow transplantation. In this population, a phase 3 randomized trial is showing a superior efficacy of letermovir compared with placebo in preventing CMV disease ^52^ with myelotoxicity and nephrotoxicity rates similar to those of placebo. Successful outcome was reported with compassionate use of letermovir in a lung transplant patient with CMV-resistant disease ^53^. Letermovir was also shown to be effective in treating CMV viremia in kidney transplant recipients ^54^, and a clinical trial comparing letermovir with valganciclovir for the prevention of CMV disease in donor-positive/recipient-negative kidney transplantation is starting (ClinicalTrials.gov identifier: NCT03443869). However, caution should be exercised in treating CMV infection with letermovir alone, as *in vitro* studies have shown rapid emergence of resistance on treatment ^55^. The lipid-conjugated analogue of cidofovir, brincidofovir, has high oral availability and less nephrotoxicity than cidofovir. Efficacy has been low in prevention in hematopoietic stem cell transplant patients, and few data are available in SOT recipients ^56^. Moreover, Faure *et al*. reported two cases of acute kidney injury in SOT patients who received brincidofovir ^57^.

## Reactivation of viral infections: role of immune monitoring

SOT patients are prone to the reactivation of viruses which are usually latent in immunocompetent people, such as herpes viruses—herpes simplex virus (HSV), varicella zoster virus (VZV), CMV, Epstein-Barr virus (EBV), and human herpesvirus 8—or polyomavirus (BK virus and less frequently JC virus), whether those viruses are already latent in the recipient or latent in the organ transplanted. In this context, immunological tools have been developed with the objective of providing a personalized assessment of the risk of reactivation ^58^.

### Cytomegalovirus

*In vitro* tests have been developed to detect the release of interferon-gamma (IFN-γ) induced by stimulation of lymphocytes by CMV antigens. The most used test is QuantiFERON-CMV assay (Qiagen Ltd.), a commercially available enzyme-linked immunosorbent based assay ^59^. Several studies showed that positivity of QuantiFERON-CMV at the end of prophylactic valganciclovir correlates with lower incidence of CMV disease ^60, 61^ and that patients with low viremia were more likely to have spontaneous clearance if they had positive QuantiFERON-CMV ^62^. However, data showing the use of this test in daily practice were missing. Recently, Kumar *et al*. reported the results of an interventional study using QuantiFERON-CMV in real-life practice ^63^. Patients were enrolled at completion of treatment for the first episode of CMV reactivation, and a QuantiFERON-CMV test was done with results available within 3 days. For patients with a positive test, no prophylaxis was given, whereas patients with negative QuantiFERON-CMV received 2 months of prophylaxis. Only one patient in the QuantiFERON-CMV-positive group had recurrence of asymptomatic CMV viremia. A large proportion with negative QuantiFERON-CMV developed recurrence while on secondary prophylaxis ^63^ *.* Further studies are warranted to define how QuantiFERON-CMV use could improve the management of CMV disease.

### Epstein-Barr virus

EBV reactivation can be associated with post-transplant lymphoproliferative disorders (PTLDs) in SOT patients. Several studies reported the feasibility of detection of EBV immune response *in vitro* by various techniques such as tetramer detection, intracellular staining, or ElisPOT ^64– 66^. The studies have shown conflicting results between immune response and its correlation with PTLD ^67– 69^. More studies are warranted to assess how immunological tools could improve the management of risk associated with EBV in transplantation.

### BK virus

Functional assays have also been developed to detect specific responses against BK virus. BK virus is a polyomavirus frequently reactivating after kidney transplantation, in urine and sometimes in the blood. This reactivation can lead to BK virus nephropathy and compromised kidney function ^70^. Schachtner *et al*. monitored BK virus-specific production of IFN-γ in kidney transplant recipients. The authors showed that a BK virus-specific response was detectable before transplant in 69% of patients. A decrease of this response at day +30 after transplantation was associated with increased risk of BK viremia, and the persistence of this specific response was associated with lower risk of reactivation ^71^. However, the appropriateness of use of the 15 peptides used in this test has been questioned, as it is known to elicit mainly a CD4 ^+^ T-cell response. Leboeuf *et al*. recently reported an immune monitoring of BK virus-specific immunity using 9mer peptides in an immunodominant epitope ^72^. They measured the cellular activity at 0, 6, and 12 months after transplant and showed that viremia was associated with an increase of specific 9mer-specific cellular response. A high response was associated with increased clearance of viremia ^72^. More studies are warranted to define its use in real-life practice for the management of patients with BK viremia.

## Immunization

### Shingles vaccines

Herpes zoster virus reactivation can affect up to 20% of SOT patients during their lifetime ^73^. Immunization pre-transplantation relies on varicella vaccine if the VZV serology is negative and shingles vaccine if the patient is seropositive. However, these vaccines are live attenuated vaccines and therefore are contra-indicated after transplant ^74^. In transplant candidates, owing to the risk of viremia after transplantation, the administration of live vaccine should be avoided if the transplant is urgent.

An inactivated vaccine against shingles was recently approved by the FDA after two randomized trials showing clinical efficacy for the prevention of shingles compared with placebo in adults more than 50 years old ^75^ and more than 70 years old ^76^. Few data exist in immunocompromised patients, and so far there has been only one immunogenicity study in patients who received autologous stem cell transplant ^77^. It is indeed a promising option for seropositive patients, but more data are required in SOT patients.

### Influenza vaccine

A yearly immunization against influenza is recommended in SOT patients, as influenza is associated with significant morbidity in those patients ^78^. The inactivated flu vaccine is recommended, but its immunogenicity is lower in these patients compared with the general population; seroconversion rates range from 15% to 70%. In the last few years, several studies reported different strategies to optimize flu immunization. Kumar *et al*. studied the effect of an adjuvanted vaccine in kidney transplant recipients and did not find significant difference in seroconversion rates ^79^. A randomized clinical trial performed on 499 solid organ transplant recipients compared the use of two doses versus one dose of inactivated influenza vaccine. The seroprotection rates were higher in patients who received two doses separated by 5 weeks ^80^. The inactivated high-dose vaccine, with a fourfold increased quantity of antigens compared with inactivated standard-dose vaccine, has been shown to induce better immunogenicity and clinical efficacy in elderly people ^81^. Natori *et al*. recently reported the results of a randomized clinical trial comparing standard- and high-dose inactivated influenza vaccine in 161 SOT patients and showed higher seroconversion rates with high-dose vaccine compared with standard-dose vaccine. Both vaccines (high and standard dose) had similar safety profiles ^82^.

### Vaccination for travelers

The increasing number of transplantations done worldwide and the improvement of transplantation outcomes lead to an increasing number of SOT recipients travelling or living in areas endemic for certain infections, raising questions about the opportunity to prevent some of those diseases with vaccination. The decision about vaccines is driven by the epidemiology of infections and the expected exposure of the patient ^83^. Some vaccines are live attenuated vaccines and subsequently are contra-indicated in SOT recipients (such as yellow fever vaccine and dengue vaccine), whereas others can be administrated to SOT patients (meningococcal vaccine, Japanese encephalitis, rabies, and tick-borne encephalitis). Of note, a recent report of three fatal cases of tick-borne encephalitis in SOT recipients, related to donor-derived infection ^84^, highlights the potential severity of this viral infection in SOT patients.

## Conclusions and perspectives

The success of organ transplantation has opened doors to new challenges in infectious diseases, which are compounded by the recognition of new transmissible organisms and multidrug-resistant organisms. However, advances in vaccination may translate into better protection for solid organ transplant recipients. In the very near future, some paradigms in the field of transplant infectious diseases will change, as progress in treating infectious diseases (for instance, hepatitis C) can have a significant impact on organ donation. The emergence of new strategies indicates not only that the development of newer antimicrobials will shape the future of organ transplantation but also that tapping into better diagnostic and prognostic immunological tools can help deliver more personalized care.
